# Perceived body size across sex and weight categories and its association with body size dissatisfaction: a cross-sectional study among early primary school children in Norway

**DOI:** 10.1186/s12889-025-22219-z

**Published:** 2025-03-31

**Authors:** Tove L. Drilen, Trine T. Eik-Nes, Ellen M. I. Ersfjord, Christian A. Klöckner, Rønnaug A. Ødegård

**Affiliations:** 1 Department of Clinical and Molecular Medicine, Norwegian University of Science and Technology, Trondheim, Norway; 2 Department of Neuromedicine and Movement Science, Norwegian University of Science and Technology, Trondheim, Norway; 3 Nord-Trøndelag Hospital Trust, Levanger Hospital, Levanger, Norway; 4 Department of Health and Nursing Sciences, University of Agder, Kristiansand, Norway; 5 Department of Psychology, Norwegian University of Science and Technology, Trondheim, Norway; 6 Centre of Obesity Research, St. Olav University Hospital, Trondheim, Norway

**Keywords:** Body image, Body size misperception, Body size dissatisfaction, Overweight, Children, Boys, Public health

## Abstract

**Background:**

Inaccurate perceptions of body size, known as body size misperception (BSM), may be linked to body size dissatisfaction (BSD) and unhealthy eating behaviours. However, these associations remain inconclusive and not fully understood in young children. This study aimed to investigate the prevalence of BSM across sex and weight categories and to further assess the association between BSM and BSD in 8–to 9-year-old children.

**Methods:**

This cross-sectional study of 209 primary school children (51% boys) from central Norway was performed during the national height and weight screening program in third grade. Researcher-assisted questionnaires and Stunkard’s figure rating scales adapted for children were used to assess two dimensions of body image: BSM (perceived-actual body size) and BSD (perceived-ideal body size). The agreement between children's ideal and actual body size was also evaluated (actual-ideal body size). Associations between BSM and BSD were examined by multinomial logistic regression, adjusting for sex, Body Mass Index (BMI), socioeconomic status, ethnicity, and residence.

**Results:**

BSM was frequently observed (81%), with most children overestimating their body size (67%). Boys tended to overestimate their body size more frequently (75% vs. 59%, *p* = 0.014) and indicated a larger mean ideal body size than girls (silhouette fig. 4.2 [95% CI 4.0, 4.5] vs. 3.9 [95% CI 3.7, 4.1], *p* = 0.012). According to BMI, overestimation was common among children with underweight (100%) and average weight (78%), whereas underestimation of body size was prevalent among children with overweight/obesity (59%). Although 23% desired at least one body size figure smaller or larger than their perceived size, interpreted as BSD, no difference was observed between mean perceived and mean ideal body size (silhouette fig. 4.1 in both groups). No significant association was found between BSM and BSD, for either underestimation (OR 1.32 [95% CI 0.33, 5.32]) or overestimation (OR 0.99 [95% CI 0.38, 2.58]) of body size.

**Conclusions:**

Boys and girls from all weight categories frequently misperceived their body size toward their ideal body size, with overestimation of underweight and average weight status and underestimation of overweight status being most frequently reported. No association was found between BSM and BSD, however, the long-term health consequences of BSM should be further elucidated.

**Supplementary Information:**

The online version contains supplementary material available at 10.1186/s12889-025-22219-z.

## Background

Body image is a multidimensional concept encompassing the mental representation of one’s body size, shape, and form [1]. The construct includes perceptions, thoughts, feelings, and behaviours associated with one’s body [2] and plays an important role in children's and adolescents' psychological health and well-being [3, 4]. Body size dissatisfaction (BSD) is the most researched construct of body image, referring to the negative subjective evaluation of one’s body size compared with ideal body size [1], which is a significant risk factor for eating disorders and poor mental health among children and adolescents [5–7]. The less investigated perceptual construct of body image, body size misperception (BSM), refers to the ability to perceive one’s actual body size and is indicated as either accurate estimation, underestimation, or overestimation of body size [8, 9].

The main concern about inaccurate perceptions of body size has been the overestimation of body size, and its potential association with unhealthy weight-control behaviours and the development of eating disorders [10, 11]. An additional concern of BSM has been related to the underestimation of body size among children categorized with overweight or obesity [8] and their parents [12], which has been considered a barrier to participation in lifestyle interventions, with a concurrent increased risk of unintentional weight gain and obesity [9, 13]. By contrast, a systematic review indicated that an accurate perception of overweight status may not necessarily lead to a healthier lifestyle but rather predict future weight gain [14]. Furthermore, emerging research suggests beneficial health effects related to the underestimation of overweight status, which may protect against the psychosocial consequences of obesity [8, 15]. For example, among adolescents and young adults, underestimation of overweight status has been associated with less weight gain [14, 16], a reduced likelihood of disordered eating behaviour [17], fewer depressive symptoms [18], higher body satisfaction [19], and better life satisfaction and mental health [20]. Among elementary school children, underestimation of overweight status has also been found to be associated with improved self-esteem [21] and lower risk of excess weight gain [14, 22]. Hence, BSM may have both positive and negative implications for children’s health.

The exact age at which children develop the cognitive ability to accurately estimate their body size remains uncertain. While some researchers suggest that body size can be accurately estimated from the age of 8 years [23], others propose it may develop as early as the preschool years [24, 25]. However, substantial variations exist across studies, with body size misperception being commonly observed among boys and girls, from all weight categories, and across ethnic minorities and socioeconomic classes [8, 26, 27]. Different patterns of BSM have previously been observed across sexes, whereby males seem to be more likely to underestimate, and females more likely to overestimate their body sizes [28]. However, findings related to sex differences in BSM among children are inconsistent [8]. Previous research has also indicated differences in the ability to perceive children’s actual body size across body mass index (BMI) categories, with misperceptions being more common among children with underweight or overweight/obesity than children with average weight [13, 29].

BSM has previously been described as a distinct construct unrelated to BSD [30]. However, emerging research suggests a potential association between these two aspects of body image in adolescents and adults [19]. While both BSM and BSD are prevalent among older children and adolescents, their relationship remains understudied in young prepubescent children [24, 31–34]. Greater insight into the impact of BSM on young children’s health is essential to fostering a healthy body image and preventing unhealthy eating behaviors, independent of BMI category. Most existing studies have been conducted in the USA and Australia, highlighting the need for more research within a European context. Hence, this study aims to explore the prevalence of BSM across sex and weight categories and its associations with BSD in a sample of 8– to 9-year-old school children in Norway. We hypothesized that there would be an association between BSM and BSD, particularly among those categorized with overweight and obesity.

## Methods

### Study design and location

This is a cross-sectional study of young children’s perceptions of body image performed in a school setting in Norway. The inclusion criteria were enrolment in third grade and participation in the national height and weight screening program, with no exclusion criteria. Data was collected between November 2021 and April 2022.

### Recruitment of participants

All 56 primary schools in two municipalities of central Norway were invited to participate. Twenty-two schools gave consent, 28 schools did not respond, and six schools refrained from participating in the study. To achieve the required sample size based on power analysis, while ensuring a diverse and representative sample, we purposively selected eight schools, comprising 310 eligible children. Selection criteria included urban and rural distribution, geographical spread, sociodemographic status, and ethnic diversity. Study information was available in three languages (English, Arabic, and Tigrinya) to promote study participation. Assent and consent were provided by 226 children and their parents/caregivers (73% agreement rate). Children who were absent on the day of data collection (*n* = 11), those who withdrew their assent (*n* = 2), and those with missing data (*n* = 4) were excluded, leaving a total of 209 children, accounting for a participation rate of 67% [35].

### Anthropometric and demographic variables

Height (cm) and weight (kg) were measured individually by a trained school nurse in a separate room, following national guidelines [36]. Children wore light clothing and no shoes, and measurements were taken using medically approved height stadiometers and digital scales to the nearest 0.1 cm and 0.1 kg, respectively. BMI (kg/m^2^) was calculated, and participants were categorized with underweight, average weight, overweight, or obesity, according to the International Obesity Task Force (IOTF) age and sex-specific BMI reference values for children [37].

Children with at least one parent from a non-Western country of origin were denoted as non-Western [38]. Socioeconomic status (SES) was categorized as low, medium, or high based on the proportion of children living in persistently low-income households within each school region. In the absence of individual SES data, SES was estimated for each school region based on the socioeconomic profile of each school’s surrounding community [39].

### Body image assessment

The method used to assess body image in this study was based on a child-friendly interview, previously described by Birbeck and Drummond [25], and a visual figure rating scale, originally developed by Stunkard [40] and adapted to 9–10-year-olds [41]. The scale was validated for children aged 7–12 years [42] and consisted of nine body silhouette figures for boys and girls, ranging from 1 (underweight) to 9 (severe obesity), with equal heights and different hair colours.

### Actual body size

**Fig. 1:**
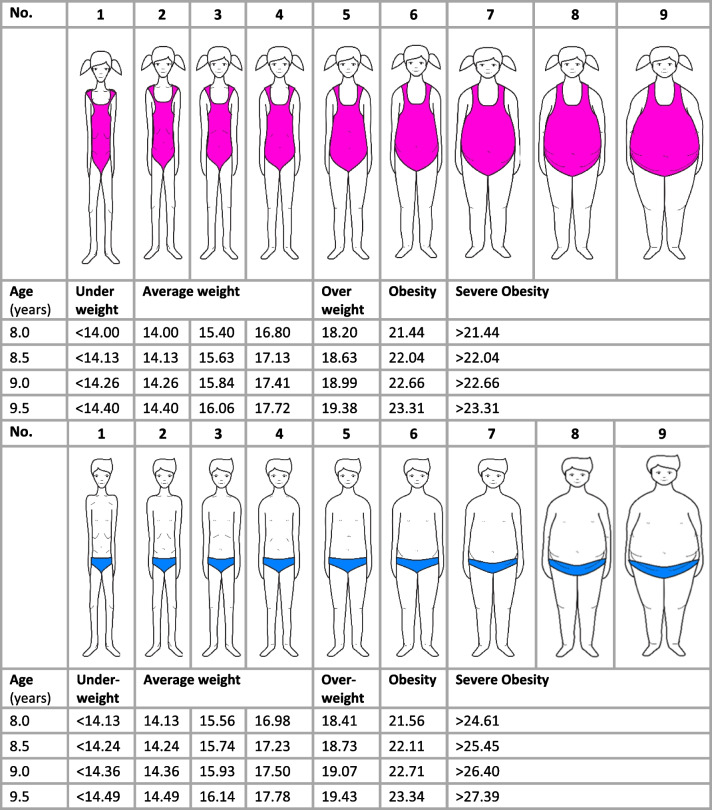
BMI cut-offs used to estimate actual body size for 8–9-year-old boys and girls. The body silhouette figures are used with permission from Dr Tiggemann

**Fig. 2:**
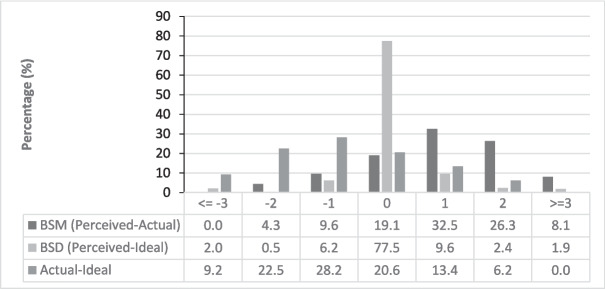
Distribution of body size misperception (BSM), body size dissatisfaction (BSD), and the discrepancy scores between actual and ideal body size

The nine body silhouettes used in this study were equally assigned the following BMI categories for girls and boys: fig. 1 (underweight), figs. 2–4 (average weight), fig. 5 (overweight), and figs. 6–9 (obesity), according to a previous study among children within the same age group [43]. BMI categories have previously been assigned to Stunkard's body silhouettes for both adults [44] and children [45]. In this study, the IOTF BMI reference cut-offs for children [37] were applied to each of the nine body size figures, as shown in Fig. 1, with corresponding cut-offs for Body Mass Index z scores (BMIz) in S-Table 2. The body figure corresponding to the child's objectively measured BMI was denoted as actual body size.

### Body size misperception (BSM)

BSM was estimated as the discrepancy between perceived and actual body size figures (perceived-actual). Negative values indicated underestimation, positive values indicated overestimation, and no discrepancy indicated accurate perceptions of body size. Children who perceived themselves at least one figure size smaller or larger than their actual body figure were denoted as having BSM.

### Body size dissatisfaction (BSD)

BSD was estimated as the discrepancy between perceived and ideal body size figures (perceived-ideal). Negative values indicated a desire for a larger body size, positive values indicated a desire for a thinner body size, and no discrepancy were denoted body satisfaction. Twenty-three percent of the children desired at least one body size figure thinner or larger than their perceived body figure, which was interpreted as BSD.

### Actual versus ideal body size

The agreement between children’s actual and ideal body size was determined as the discrepancy between their ideal and actual body size figure (actual-ideal). Negative values indicated an ideal figure size larger than the actual figure size, and positive values indicated an ideal body size figure thinner than the actual body size figure. No discrepancy indicated agreement between actual and ideal body size.

### Procedure

The school nurse collected information about the children’s date of birth and ethnicity and measured their height and weight. In a private classroom, individual questionnaires were performed by experienced researchers and undergraduate students trained in body image assessment. The questionnaire was previously published as part of another study including the same participants [35]. Paper, coloured pencils, and fidget toys were used to create a safe and child-friendly atmosphere. All the children were initially informed about confidentiality and their right to withdraw at any time.

To avoid potential bias, each body silhouette was presented randomly for each child on separate A4 sheets with numbers hidden on the back of the paper [46]. To identify perceived and ideal body size figures, the following questions were asked (S-Table 1):

1. “Which of the pictures do you think looks the most like you?” (Perceived body size)
2. “If I was a magician with a magic wand and could transform you into one of these pictures, would you choose to stay the same or be transformed into one of the other body figures?”

Those who indicated no desire for a different body size figure were categorized as ‘satisfied’ with their perceived body size. Children who reported an ideal body size thinner or larger than their perceived body size figure were categorized as ‘dissatisfied’ with their perceived body size and further asked the following questions:

3. “Which of these figures would you like to be transformed into? Why? Remember, you may have to stay that way for a long time, so it needs to be a body figure that you like” (Ideal body size).

Three discrepancy scores were estimated based on children's actual, perceived, and ideal body size figures: 1) Perceived-actual (BSM), 2) Perceived-ideal (BSD), and 3) Actual-ideal body size figures.

### Statistical analyses

IBM SPSS Version 29 was used for statistical analysis. The overweight and obesity categories were merged due to few children with obesity (*n* = 3). Moreover, children attending schools categorized with high and medium SES were merged in the statistical analysis and compared with those from schools with low SES.

Mean body size figures were estimated for the children’s (1) Actual, (2) Perceived, and (3) Ideal body sizes and compared across sexes, BMI categories, SES, ethnicity, and residency. Paired samples t tests were used to analyse differences between actual, perceived, and ideal body size figures, whereas differences across sexes and demographics were analysed via independent samples t tests. One-way ANOVA was used to study differences between BMI categories (underweight, average weight, and overweight/obesity) in terms of actual, perceived, and ideal body size.

Underestimation, accurate estimation, or overestimation of body sizes are presented as percentages and numbers, % (n). Differences in the correct estimation, underestimation, and overestimation of body size were analysed separately via chi-square tests across sex, BMI categories, and demographic variables. Multinomial regression analyses were used to estimate the associations between BSM (under- or overestimation relative to correct estimation) and BSD (desire for a thinner/larger body size or no BSD), adjusting for sex, BMI, SES, ethnicity, and urban/rural residence.

This study is a post hoc analysis of a previous study using body size dissatisfaction (BSD) as the primary outcome, with a predetermined sample size of 134 based on a 30% BSD prevalence, a 0.05 margin of error, and 80% statistical power [35]. The present study applied BSM as the primary outcome, and a power calculation based on 80% actual rates of BSM, with equal statistical assumptions, found 117 participants to be sufficient.

Due to the exploratory nature of our study, we defined a statistical significance level of *p* < 0.05, however, p-values between 0.05 and 0.01 were interpreted with caution.

## Results

### Participant characteristics

The study sample comprised 209 children (51% boys) aged 7.9–9.3 years from central Norway, including 13 children with underweight (6%), 157 with average weight (75%), 36 with overweight (17%), and 3 with obesity (1%). Furthermore, 29 of the children were of non-western origin (14%), 105 went to school in a region categorized with low SES (50%), and 53 attended rural schools (25%). No differences were found between participants and non-participants (*n* = 84), except for ethnicity. However, the higher proportion of children of non-Western origin among non-participants was aligned with the regional average [47], indicating a representative sample of 8- to 9-year-old children from central Norway.

### Actual, perceived, and ideal body size

Based on the nine body silhouettes, mean values for children’s actual, perceived, and ideal body size figures are presented in Table 1. Children’s mean actual body size figures were almost identical across the sexes. Compared to girls, boys indicated a larger perceived body size (silhouette fig. 4.3 vs. 3.9, Cohen’s d = 0.49, 95% CI [0.21, 0.76], *p* < 0.001) and a larger ideal body size (silhouette fig. 4.2 vs. 3.9, Cohen’s d = 0.35, 95% CI [0.08, 0.62], *p* = 0.012). The mean ideal body size figure did not differ across BMI categories; however, children with overweight/obesity tended to perceive their body size to be larger than their peers with average weight (silhouette fig. 4.4 vs. 4.0, Eta-squared (η^2^) = 0.03, 95% CI [0.00, 0.09], *p* = 0.028). Perceived body size was correlated with both ideal body size (r = 0.59, *p* < 0.001) and actual body size (r = 0.25, *p* < 0.001), whereas actual and ideal body size was unrelated (r = 0.08).

**Table 1:** Children's mean actual, perceived, and ideal body size figures, according to sex, BMI, and demographics

	**Actual body size figure ** Mean [95% CI]	**Perceived body size figure ** Mean [95% CI]	**Ideal body size figure ** Mean [95% CI]
All children (*n* = 209)	3.2 [3.0, 3.3]	4.1 [4.0, 4.2]	4.1 [3.9, 4.2]
**Sex**			
Girls (n=102)	3.2 [3.0, 3.5]	3.9 [3.8, 4.1]	3.9 [3.7, 4.1]
Boys (n=107)	3.1 [2.9, 3.4]	4.3 [4.1, 4.5]	4.2 [4.0, 4.5]
**BMI category**			
Underweight (n=13)	1.0 [1.0, 1.0]	4.0 [3.4, 4.7]	4.0 [2.8, 5.2]
Average weight (n=157)	2.9 [2.8, 3.0]	4.0 [3.9, 4.2]	4.0 [3.8, 4.2]
Overweight/obesity (n=39)	5.1 [5.0, 5.2]	4.4 [4.1, 4.7]	4.4 [4.1, 4.7]
**Socioeconomic status**			
Low (*n* = 105)	3.0 [2.8, 3.3]	4.1 [3.9, 4.2]	3.9 [3.7, 4.1]
Medium/high (*n* = 104)	3.3 [3.0, 3.6]	4.2 [4.0, 4.4]	4.2 [3.9, 4.4]
**Ethnicity**			
Non-Western (*n* = 29)	3.1 [2.5, 3.6]	4.0 [3.68, 4.4]	4.0 [3.5, 4.5]
Western (*n* = 180)	3.2 [3.0, 3.4]	4.1 [4.0, 4.3]	4.1 [3.9, 4.2]
**Residence**			
Rural (*n* = 53)	3.3 [3.0, 3.7]	4.2 [4.0, 4.4]	4.2 [3.9, 4.4]
Urban (*n* = 156)	3.1 [2.9, 3.3]	4.1 [3.9, 4.2]	4.0 [3.8, 4.2]

Mean values are based on Stunkard's figure rating scale consisting of nine body silhouettes, ranging from 1 (underweight), 2–4 (average weight), 5 (overweight) to 6–9 (obesity)

*Abbreviations*: *BMI* Body Mass Index, *CI* confidence interval

### Body size misperception

A significant difference between mean actual and mean perceived body size was found (silhouette fig. 3.2 vs. 4.1, Cohen’s d = −0.72, 95% CI [−0.87, −0.57], *p* < 0.001) (Table 1). As shown in Table 2, most children (81%) misperceived their body size with at least one body size figure, including 67% who overestimated and 14% who underestimated their body size. Differences in BSM rates were observed across the sexes, with more boys than girls tending to overestimate body size (75% vs. 59%, *p* = 0.014). According to BMI categories, all children with underweight and most of the children with average weight (78%) overestimated their body size, whereas more than half of children with overweight/obesity (59%) underestimated their body size. Furthermore, a higher proportion of children with overweight/obesity perceived their body size figure more accurately (31%) than children from both underweight (0%) and average weight (18%) categories.

**Table 2:** Variation in body size perception according to sex, Body Mass Index, and demographics

	**Correct estimation **	**Underestimation**	**Overestimation**
**Total % **(*n* = 209)	19.1% (*n* = 40)	13.9% (*n* = 29)	67.0% (*n* = 140)
**Sex (%)**			
Girls (*n* = 102)	23.5% (*n* = 24)	17.6% (*n* = 18)	58.8% (*n* = 60)
Boys (*n* = 107)	15.0% (*n* = 16)	10.3% (*n* = 11)	74.8% (*n* = 80)*
**Body Mass Index**			
Underweight (*n* = 13)	0.0% (*n* = 0)	NA	100.0% (*n* = 13)
Average weight (*n* = 157)	17.8% (*n* = 28)	3.8% (*n* = 6)	78.3% (*n* = 123)
Overweight/obesity (*n* = 39)	30.8% (*n* = 12)*	59.0% (*n* = 23)**	10.3% (*n* = 4)**
**Socioeconomic status**			
Low (*n* = 105)	20.0% (*n* = 21)	13.3% (*n* = 14)	66.7% (*n* = 70)
Not low (*n* = 104)	18.3% (*n* = 19)	14.4% (*n* = 15)	67.3% (*n* = 70)
**Ethnicity**			
Western (*n* = 180)	18.3% (*n* = 33)	14.4% (*n* = 26)	67.2% (*n* = 121)
Non-western (*n* = 29)	24.1% (*n* = 7)	10.3% (*n* = 3)	65.5% (*n* = 19)
**Residency**			
Urban (*n* = 156)	17.3% (*n* = 27)	14.1% (*n* = 22)	68.6% (*n* = 107)
Rural (*n* = 53)	24.5% (*n* = 13)	13.2% (*n* = 7)	62.3% (*n* = 33)

Results are presented as percentages and numbers, % (*n* =)

Chi-square tests with post hoc comparison assessed differences in correct estimation, underestimation, and overestimation of body size across sex, Body Mass Index, and demographic variables

Level of significance: * *p* < 0.05, ** *p* < 0.001

*NA* not applicable

The range of BSM is illustrated in Fig. 2, with perceived body size varying from two figures smaller to five figures larger than actual body size, including over one in three children (39%) reporting at least two body size discrepancies. According to BMI categories, all children with underweight, 26% of children with average weight, and 54% of children with overweight/obesity misperceived their body size with at least one category (Table 3).

**Table 3:** Agreement between perceived and actual weight categories

	PERCEIVED WEIGHT CATEGORIES		
ACTUAL WEIGHT CATEGORIES	Underweight (n = 1)	Average weight (n = 146)	Overweight/obesity (n = 62)
Underweight (n = 13)	0% (n=0)	69% (n = 9)	31% (n = 4)
Average weight (n = 157)	1% (n = 1)	74% (n=116)	25% (n = 40)
Overweight/obesity (n = 39)	0% (n = 0)	54% (n = 21)	46% (n=18)

The weight categories included underweight (silhouette fig. 1), average weight (silhouette figs. 2–4), overweight (silhouette fig. 5), and obesity (silhouette figs. 6–9)

Data are presented as % (n = ), with agreements between weight categories highlighted

### Body size dissatisfaction

As shown in Fig. 2, one in four (23%) children indicated BSD by selecting an ideal body size at least one body size figure different from their perceived body size figure, however, no difference was observed between mean perceived and ideal body size (silhouette fig. 4.1 in both groups) [35]. BSD varied from the desire to be four figures smaller to five figures larger than the perceived body size. However, less than 10% indicated more than one discrepancy between perceived and ideal body size.

### Actual versus ideal body size

A significant difference was found between the mean actual and ideal body size (silhouette fig. 3.2 vs. 4.1; Cohen’s d = −0.56, 95% CI [−0.71, −0.42], p < 0.001) (Table 1). Most children (80%) indicated an ideal body size of at least one figure different from their actual body size, with 60% indicating a larger ideal and 20% indicating a smaller ideal body size compared to their actual body size Fig. 2. Boys' ideal body size deviated more from their actual body size than girls did. The discrepancy between children's actual and ideal body sizes ranged from −8 to + 2.

### Associations between BSM and BSD

No associations between BSM and BSD were found for either underestimation (OR 1.32 [95% CI 0.33, 5.32]) or overestimation of body size (OR 0.99 [95% CI 0.38, 2.58]) after adjusting for sex, BMI, and demographic variables (Table 4). These findings were substantiated by a sensitivity analysis utilizing discrepancy scores of ± 2 body size figures in the assessment of BSM.

**Table 4:** Multinomial logistic regression for the association between BSM and BSD adjusted for confounders

	**Body size misperception (BSM)**	
	**Underestimation** OR [95% CI]	**Overestimation** OR [95% CI]
**Body size dissatisfaction (BSD)** (Ref: BSD)	1.32 [0.33, 5.32]	0.99 [0.38, 2.58]
Sex (Ref: Boys)	2.03 [0.60, 6.88]	0.37 * [0.17, 0.83]
BMI Overweight (Ref: Average weight)	**12.53** ** [3.45, 45.56]	**0.06** ** [0.02, 0.21]
Socioeconomic status (Ref: Low SES)	0.68 [0.19, 2.43]	1.73 [0.75, 3.96]
Ethnicity (Ref: Non-Western)	1.90 [0.38, 9.49]	1.03 [0.35, 3.05]
Residence (Ref: Rural)	1.71 [0.46, 6.32]	1.41 [0.58, 3.44]

Data are presented as Odds ratio (OR) with a 95% confidence interval (CI)

Dependent variable: Body size misperception (underestimation and overestimation) compared to correct estimation. Independent variable: Body size dissatisfaction (reference: BSD)

Level of significance: * *p* < 0.05, ** *p* < 0.001

## Discussion

In this study among 8–to 9-year-old Norwegian school children, inaccurate perceptions of body size were highly prevalent. Although most children overestimated their body size, particularly boys, underestimation was frequently reported among those with overweight and obesity. In contrast with our hypothesis, there was no association between body size misperception (BSM) and body size dissatisfaction (BSD). BSM was prevalent among 81% of the children in this study, which is consistent with other studies with comparable age groups and similar methodologies [32, 48–50]. This finding reinforces that young children misperceive their body size across sexes, weight categories, ethnicities, and socioeconomic classes.

The inaccurate perceptions of body size observed among the participants in this study may be explained by several factors. As earlier proposed, the participants’ ability to assess their body size may have been influenced by their cognitive and communicative abilities [25]. However, the overall bias towards overestimating body size observed in our study contrasts with most previous studies, in which children of similar age are found to underestimate body size [22, 32]. Our findings may reflect a shift from thin ideals toward larger, more muscular, and toned Westernized body ideals [51], especially among boys. Increased social media use among younger children, particularly during the COVID-19 lockdown, and greater exposure to muscular body appearance ideals as observed among adolescents [52] may have influenced children’s perceptions of body size [53].

Another possible explanation for the frequent overestimation of body size may be due to methodological differences in body image assessment. It has been suggested that studies using ascending figure rating scales (from thinness to obesity) might increase the likelihood of underestimating body size in children, as they can visually compare themselves to the smaller figures on the scale [46]. Interestingly, a study conducted in Brazil [48] utilizing a similar methodology as ours with silhouettes presented randomly on separate cards also reported an overestimation of body size, in line with findings of preadolescent children [54], suggesting that young children may have a general propensity to overestimate their body size. Furthermore, the greater number of silhouettes used in our study and the study from Brazil (9–11 figures), compared with other studies (3–7 figures) may have influenced the results. However, the available literature on psychometric properties of figure rating scales among children is inconclusive [46, 55], and it is uncertain how randomly presented figure rating scales or a greater range of body image figures may affect the rates and direction of BSM among children.

A systematic review showed that rates of misperception of body weight may be influenced by weight category and demographics, with higher rates of BSM being found in children with underweight or overweight/obesity compared to normal-weight, among lower-income households, and non-Caucasian individuals [8]. In our study, BSM was prevalent across all BMI categories, with a misperception of body size toward average observed among most children outside the average BMI category, as indicated by frequent overestimation of underweight status and underestimation of overweight status. This tendency to misperceive body size as average corresponds to several previous studies among young children [13, 28, 31, 56, 57], and may be explained by children’s desire to be ‘normal’ or have a body size ‘about right’.

Moreover, the frequent underestimation of overweight status could be explained by visual adaptation to more individuals with obesity in society [27, 58, 59], or the unawareness of having overweight, as previously demonstrated in other studies [27, 57–59]. Interestingly, one-third of children with overweight or obesity accurately estimated their body size without indicating a desire for another body size. These results contrast with most previous studies reporting high rates of BSD among children with overweight and obesity [60] and our findings may reflect a shift towards acceptance and satisfaction with having a larger body size Fig. [59].

The high rates of overestimating body size in combination with body satisfaction found across the whole sample, corresponds to a study among preschoolers [24] and preadolescent children [32], but contrast with most previous research among adolescents and adults, linking body size overestimation to body dissatisfaction [61], unhealthy weight loss behaviours [62], and eating disorders [11]. The few studies that have investigated the association between BSM and BSD among young children are inconsistent, suggesting that BSM may be either predictive [31, 34], protective [32, 63], or unrelated to BSD [24, 32]. Furthermore, the lack of association between BSM and BSD observed in our study may suggest that young children may be less emotionally affected by social norms about body size than older children and adolescents. Whether underestimation of overweight status may protect children from future BSD needs to be investigated in a longitudinal study with a larger sample.

### Implications for healthcare services

This study underscores that young children often perceive their body size differently from their actual body size or the BMI categories used by healthcare professionals. This misalignment has important implications for health communication regarding children's weight status, particularly in the context of school-based screenings and obesity prevention programs.

Healthcare services should approach communication about children’s weight with caution. The goal should be to ensure children maintain a healthy body, focusing on overall health rather than on specific weight categories and correction of BSM. Additionally, increased efforts to safeguard children’s body image should be integral to all weight-related interventions, with focus on overall health promotion and body functionality.

### Strengths and limitations

The strength of the current study is the thorough face-to-face assessment of several constructs of body image via validated and child-friendly visual methods, with several efforts to reduce bias in a diverse sample of young elementary school children. The objective measures of height and weight and similar demographic properties among participants and nonparticipants may have further strengthened the reliability of our findings.

The present study should, however, be interpreted with some limitations in mind. First, the figure rating scales used in this study considered only two dimensions of body size, thinner or larger, with no option to indicate a more muscular body size. Second, the study was limited by the small sample of children with underweight and obesity, precluding targeted analysis of these subgroups. Third, SES was categorized based on the socioeconomic profile of each school region rather than individual data. Finally, the use of only one body silhouette discrepancy and not asking the children directly about BSD cannot exclude the possibility of false positive results.

## Conclusions

More than 3 in 4 children of both sexes and from all weight categories misperceived their body size, with overestimation of underweight and average weight status and underestimation of overweight status being most frequently reported. Regardless of direction, most children misperceived their body size towards their ideal, and the level of BSD was modest. No association was found between BSD and BSM, however, the long-term health consequences of BSM should be further elucidated.

## Supplementary Information

Additional file 1.

## Data Availability

The datasets used and/or analysed during the current study are available from the corresponding author upon reasonable request and will be available in a repository.

## Abbreviations

BMI: Body Mass Index
BMIz: Body Mass Index z score
BSD: Body size dissatisfaction
BSM: Body size misperception
CI: Confidence interval
IOTF: International Obesity Task Force
OR: Odds Ratio
SES: Socioeconomic status
