# Supplementary material for: Perceived body size across sex and weight categories and its association with body size dissatisfaction: a cross-sectional study among early primary school children in Norway
Source: BMC Public Health. 2025 Mar 31;25:1210. doi: 10.1186/s12889-025-22219-z (PMC11956423; doi:10.1186/s12889-025-22219-z)
Supplement: Supplementary file 1 — Additional file 1. [file 12889_2025_22219_MOESM1_ESM.pdf]

Supplementary materials

S-Table 1: Interview Guide

Introduction

“Hello (name of child). My name is (name of researcher), and I am a researcher at Norwegian University of Science and Technology. We are trying to learn more about what children think about their bodies and health. But as adults, we know very little about what children think, and that's why we are glad that you want to be a researcher with us today. I am going to ask you some questions, and you decide what you want to answer. If you don't want to continue, just let me know, and I will bring you back to your class.”

“If you find a question difficult, you can say "I don't know" or ask us to explain. There is no right or wrong answer; the most important thing is that you tell us what you think. Sometimes it can be hard to find the right words, so we have used smiley faces that you can point to, to make it easier for you to answer.”

“We want to record our conversation so that we remember better what you said, but nobody else will hear the recording, and we will delete it when we finish our research. Any questions so far?”

Part 1: Height and weight screening (results from this part is published elsewhere)

Now, (name of school nurse) has just measured your height and weight. There are some things that we are curious about, and we hope you can help us.

1. *"How did you feel when the school nurse (name of school nurse) measured your height?" (Indicate with arms and specify that it is about height)*

You may respond by choosing one of the three faces (move your index finger over the faces):

- The first face means don't like.
- The middle one means something in between, or neither like nor dislike.
- The last one means like.

“Can you point to the smiley face that fits best with your experience with height screening?

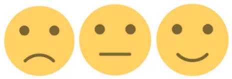

"Why did you choose this face?"

2. *"How did you feel when the school nurse (use name) weighed you?"*
   (Specify that this is about measuring body weight, on a scale).

Point to the face that fits best.

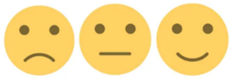

"Why did you choose this face?"

3. *"Why do you think children`s height and weight are being measured at school?"*
   (There is no right or wrong answer.)
4. *"When the students are being measured next year, do you think they should do it the same way, or do you have any suggestions for something that can be done differently?"*

Part 2: Body image

“*Can you tell me the color of your hair*?” (Get the correct set of body figure images).

Spread the nine different silhouette drawings of bodies from underweight to severe obesity randomly on the floor, with numbers from 1 to 9 on the back. Make sure the child sees them.

“Now, we are going to play a "mind game". These are pictures of children who are the same age as you, but all the pictures are a bit different. I don't know if you noticed, but they are all the same height (indicate by pointing at the head and feet), but they have different sizes. Do you see that?

5. *“Which of the pictures do you think looks the most like you?”*

"If I was a magician with a magic wand and could transform you into one of these pictures, would you choose to stay the same or be transformed into one of the other body figures?”

6. *If yes, which of these figures would you like to be transformed into? Why?*

S-Table 2: Body Mass Index z-score cut-offs corresponding to the nine silhouette figures

**Silhouette number**	**Body Mass Index z-score** (BMIz)
1 (Underweight)	-1.00 > BMIz
2 (Average weight)	-1.00 ≤ BMIz < -0.33
3 (Average weight)	-0.33 ≤ BMIz < +0.34
4 (Average weight)	+0.34 ≤ BMIz < +1.00
5 (Overweight)	+1.00 ≤ BMIz < +2.00
6 (Obesity)	+2.00 ≤ BMIz < +3.00
7-9 (Severe obesity)	+3.00 ≤ BMIz
